# Primary mediastinal yolk sac tumor: A case report and literature review

**DOI:** 10.1002/ccr3.7781

**Published:** 2023-08-08

**Authors:** Diqing Wu, Kun Zhang, Xueqin Zhang

**Affiliations:** ^1^ Department of Cardiac and Macrovascular Surgery Suzhou Hospital of Anhui Medical University Suzhou China; ^2^ Department of Oncology Suzhou Hospital of Anhui Medical University Suzhou China

**Keywords:** case reports, literature review, primary mediastinal yolk sac tumor, prognosis, treatment

## Abstract

**Key Clinical Message:**

There are limited published cases of primary mediastinal yolk sac tumor (PMYST), with no consensus on the best treatment alternative. By far, the surgery oriented comprehensive therapies are the main treatment methods. The surgical strategy should be individualized and aimed at radical resection, considering all the possibilities, including the use of cardiopulmonary bypass and prosthetic materials.

**Abstract:**

A 15‐year‐old boy was diagnosed as PMYST. The tumor, with a size about 13 × 12 × 8 cm, was located in the right upper mediastinum, closely adhering to ascending aorta, superior vena cava, right atrium, and the right hilum. After 6 cycles chemotherapy of bleomycin, etoposide, and cisplatin (BEP), no significant change was found in the size of tumor. Subsequently, an extended tumor excision including partial resection of the right lung, the pericardium, the diaphragm and the right phrenic nerve, was performed successfully with cardiopulmonary bypass on standby. During 6 months of follow‐up, there was no tumor recurrence. Meanwhile, in PubMed, we searched the English case reports and case series of PMYST during the past decade. A total of 73 articles were retrieved, in which 22 articles on the therapy and prognosis of PMYST were extracted and reviewed, included 16 case reports and 6 case series with a total of 52 patients. Due to the rarity of PMYST, it is difficult to provide a specific treatment regimen. The surgery‐oriented comprehensive therapies are still the main treatment methods. The surgical strategy should be individualized and aim at radical resection, considering all the possibilities, including the use of cardiopulmonary bypass and prosthetic materials.

## 1 INTRODUCTION

Mediastinal germ cell tumors (MGCTs) are the most common extragonadal germ cell tumors, in which yolk sac tumor (YST) is one of the histological subtypes occurring mostly in young males,^1^ with the incidences of 12% in children (0–14 years old), 73.5% in adolescents and young adults (15–39 years old).^2^ But in fact, primary mediastinal yolk sac tumor (PMYST) is extremely rare. In terms of the primary site, it only accounted for 15.4% of the overall incidence of YST.^3^ Besides, PMYST is difficult for an early diagnosis, and usually was found for the symptoms of compression and/or fever when it has grown significantly. Markedly elevated AFP level is the typical clinical characteristic. Histopathology and immunohistochemistry are the gold standards for a definite diagnosis.^4^

In this article, we report a 15‐year‐old patient of PMYST and review relevant literatures published in PubMed during the past decade, with the purpose of providing some experience and insight for the clinical treatment of this rare malignancy.

## 2 CASE PRESENTATION

**FIGURE 1:**
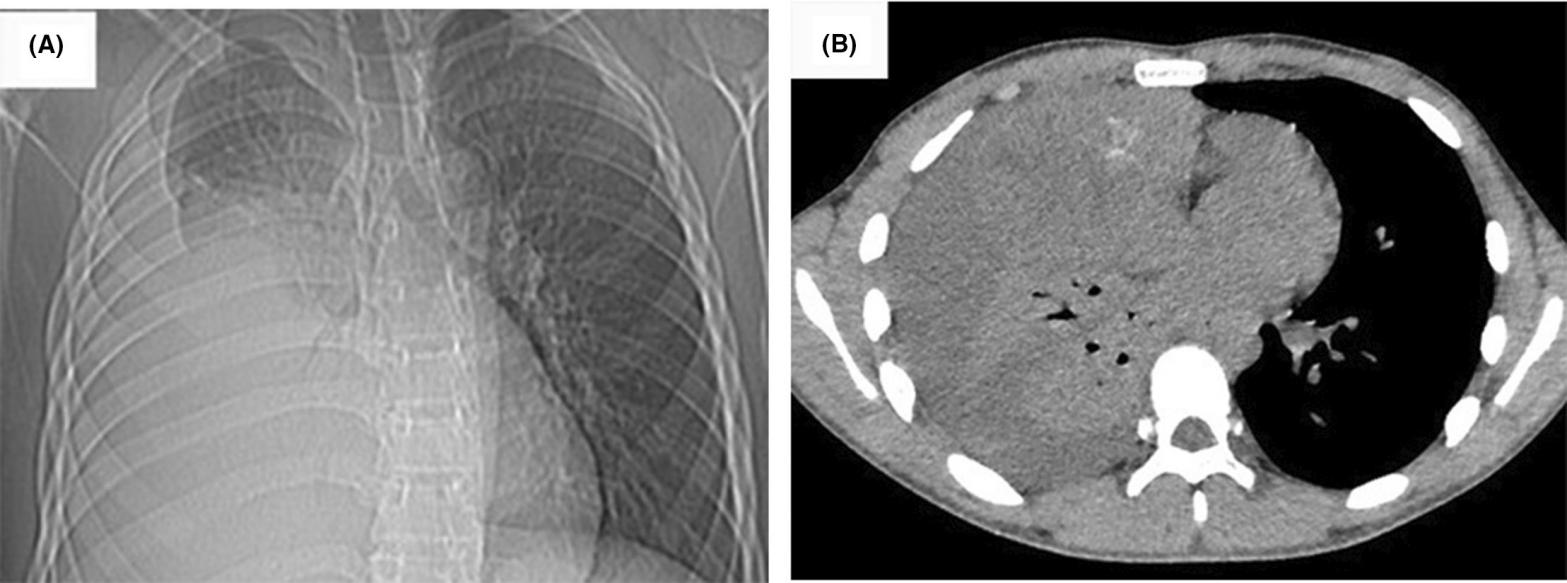
(A) Anterior–posterior chest x‐ray and (B) computed tomography scan of the chest: a huge tumor of mediastinum and severe right pleural effusion were first discovered.

**FIGURE 2:**
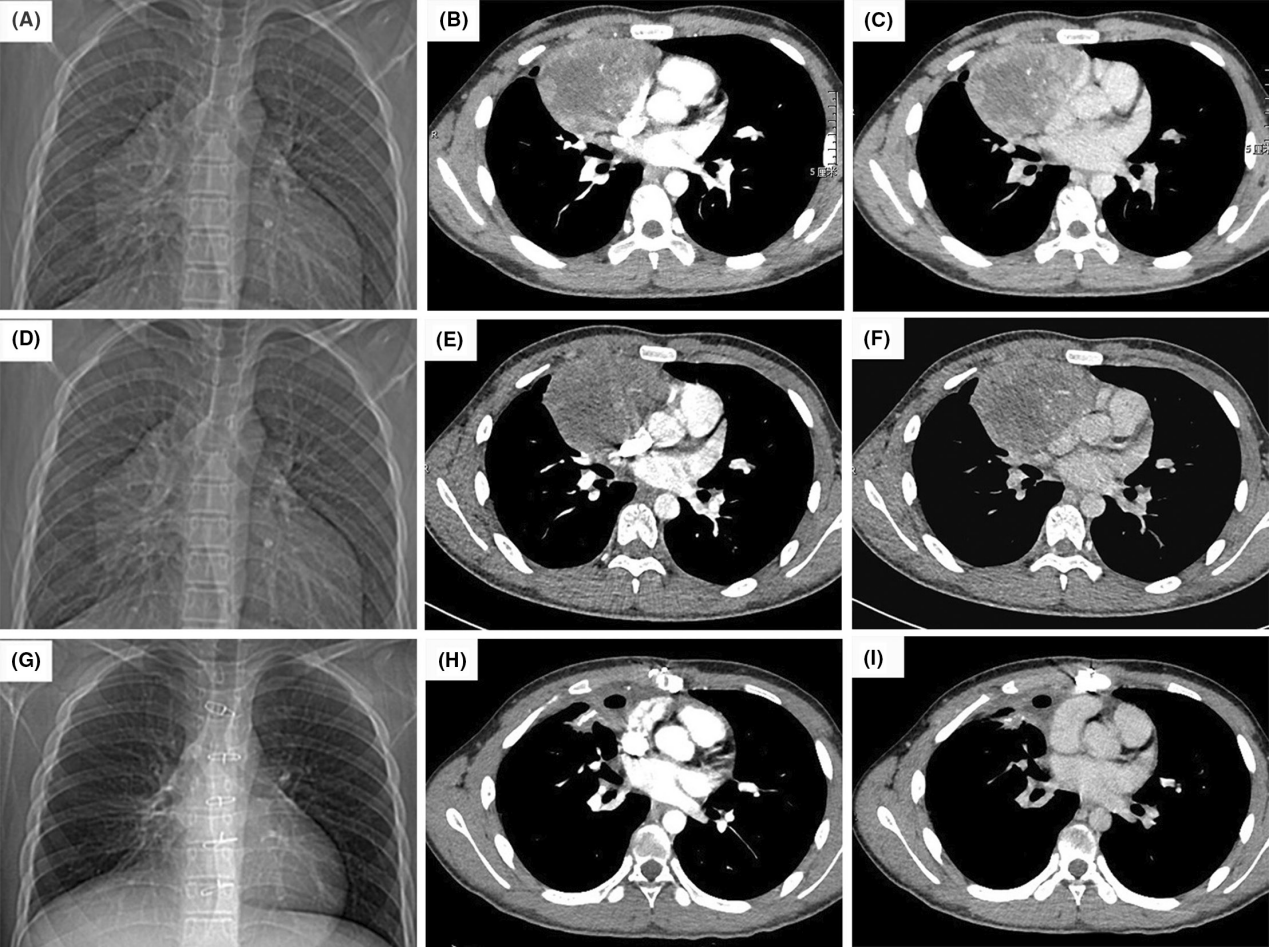
(A) Anterior–posterior chest x‐ray and (B, C) chest computed tomography scans of arterial and venous phase: a huge tumor of mediastinum before treatment; (D) anterior–posterior chest x‐ray and (E, F) chest computed tomography scans of arterial and venous phase: the size of the tumor was not significantly changed after 6 cycles of chemotherapy; (G) anterior–posterior chest x‐ray and (H, I) chest computed tomography scans of arterial and venous phase: no sign of tumor recurrence 6 months after surgery.

A 15‐year‐old male adolescent was diagnosed as PMYST by needle biopsy in another hospital 2 months before the admission to our hospital. Reviewing the initial history, the tumor was discovered accidentally for the symptoms of chest tightness, shortness of breath and fever. The initial chest computed tomography (CT) showed complete right lung atelectasis, severe right pleural effusion, and large anterior mediastinal space occupying lesion (Figure 1A,B). The symptoms relieved quickly following the administration of antibiotics and closed thoracic drainage, through which about 2000 mL hemorrhagic fluid was drained out. Afterward, the needle biopsy was performed, and PMYST was diagnosed based on the results of immunohistochemistry combined with hematoxylin–eosin (HE) staining. Then, he was referred to the oncology department of our hospital for chemotherapy, and a BEP regimen (bleomycin, etoposide, and cisplatin) was adopted. The pre‐chemotherapy enhanced CT showed that the tumor was located in the right upper mediastinum with a size of 13 × 12 × 8 cm, and closely adhered to the ascending aorta, the superior vena cava, the right atrium, and the right hilum. The external of the right atrium had been seriously compressed (Figure 2A–C).

After 6 cycles of chemotherapy of BEP, he was admitted to our department for further surgical treatment. Compared to the state of pre‐chemotherapy, no significant change was found in the size of the tumor on the reexamined enhanced chest CT image (Figure 2D–F), and the serum AFP was persistently above 1210 μg/L, maintained at the level of pre‐chemotherapy. In addition, no evidence of tumor metastasis was found in preoperative examination results. Considering all the possibilities during operation, a predesigned operative programme was made, including the use of cardiopulmonary bypass (CPB), and prosthetic vessel replacement. The operation was performed via median sternotomy. Intraoperative exploration revealed that the extent of tumor invasion involved the right anterior chest wall, the whole right mediastinal pleura, party of the middle lobe of the right lung, most of the right pericardium, the top of the right diaphragm, the right pulmonary hilum, the aortic root, and proximal superior vena cava. The whole tumor, with widespread nourishing vessels covered, adhered extensively to the surrounding tissues. Especially, in the middle and lower segments, the tumor completely invaded into the diaphragm, lung lobe, and pericardium. After sufficient dissociation, an extended excision procedure was performed, including the whole tumor resection, the right mediastinal pleurectomy, thymectomy, partial pericardiotomy and patch repair, pulmonary wedge resection, partial diaphragm resection, partial phrenic nerve resection and anastomosis. CPB and prosthetic vessel were not used. The total volume of intraoperative bleeding was about 250 mL.

**FIGURE 3:**
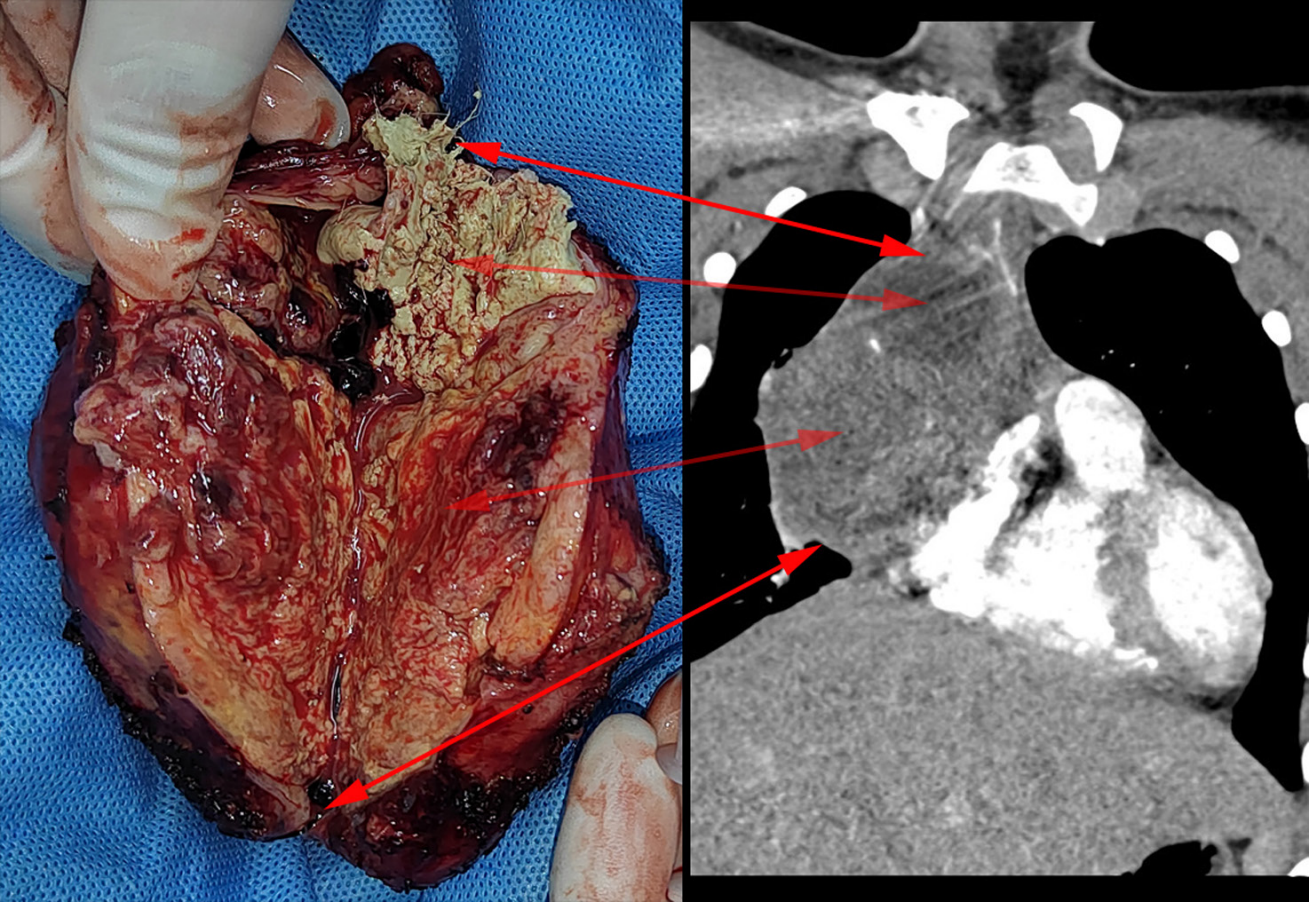
Gross examination of resected specimens: the tumor demonstrated a cystic and solid mixed lesion. The superior segment of the tumor mixed with sebaceous, hair and fat. The lower segment of the tumor showed pale‐gray, gray‐white, solid, soft, and tough tissue characterizations in different areas with local hemorrhage necrosis to different degrees. Both ends of the arrow indicate the position of the specimen corresponding to the image.

The macroscopic features inside the tumor demonstrated a cystic and solid mixed lesion, with sebaceous, hair, and fat mixed in the cystic lesion of the superior segment. The morphology on the cut surfaces of the lower segment, showed pale‐gray, gray‐white, solid, soft, and tough tissue characterizations in different areas with local hemorrhage necrosis to different degrees (Figure 3). The immunohistochemical results showed that CK (++), AFP (+), SALL‐4 (++), GPC‐3 (+), CD117 (++), Vim (−), PLAP (−), OCT3/4 (−), CD30 (−).

The patient recovered smoothly without diaphragmatic paralysis and was discharged successfully on the seventh postoperative day. Before discharge the serum AFP was decrease to 1130 μg/L. In addition, during 6 months of follow‐up, the serum AFP was stable at normal level. The reexamined CT proved well recovered right thoracic volume and lung function without tumor recurrence (Figure 2G–I).

## 3 LITERATURE REVIEW

In PubMed, the English case reports and case series of PMYST during the past decade were searched. The following key words were used for the database search: “yolk sac tumor,” “mediastinum,” “mediastinal.” A total of 73 articles were retrieved, in which 22 articles on the therapy and prognosis of PMYST were extracted, including 16 case reports and 6 case series, involving 52 patients in total.^5^, ^6^, ^7^, ^8^, ^9^, ^10^, ^11^, ^12^, ^13^, ^14^, ^15^, ^16^, ^17^, ^18^, ^19^, ^20^, ^21^, ^22^, ^23^, ^24^, ^25^, ^26^ Out of all the patients, only six female cases were reported. The ages of all the patients ranged from 1 to 73 years old with a mean age of 23.64 years. The reported maximum diameter of the tumor was 17 cm with a mean diameter of 9.7 cm. And the pathological types were pure YST in 45 patients (86.54%) and mixed germ cell tumor containing YST in 7 patients (13.46%).

**FIGURE 4:**
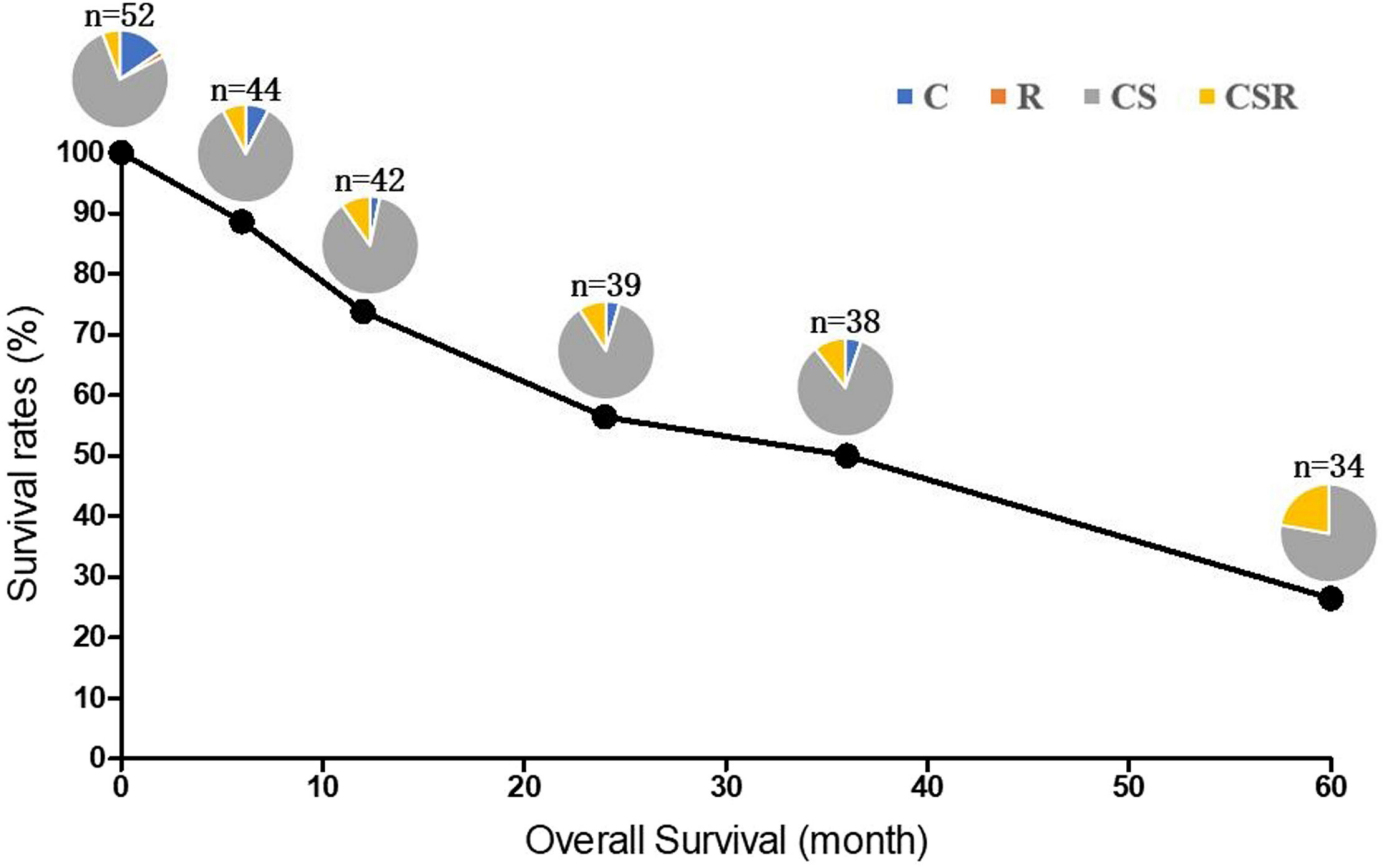
The follow‐up rates at 6 months, 1 year, 2 years, 3 years, and 5 years were 84.6%, 80.8%, 75.0%, 73.1%, and 65.4%, respectively. The survival rates at 6 months, 1 year, 2 years, 3 years, and 5 years were 88.6%, 73.8%, 59.0%, 52.6%, and 29.3%, respectively. Pie charts indicate the proportion of treatment protocols in each follow‐up period; C, chemotherapy; CS, chemotherapy + surgery; CSR, chemotherapy + surgery + radiation; *n* = the number of patients in each follow‐up period; R, radiation.

Excluded the cases who were lost to follow‐up and whose survival time was unknown, the follow‐up rates at 6 months, 1 year, 2 years, 3 years, and 5 years were 84.6%, 80.8%, 75.0%, 73.1%, and 65.4%, respectively. The pooled survival rates at 6 months, 1 year, 2 years, 3 years, and 5 years were 88.6%, 73.8%, 59.0%, 52.6%, and 29.3%, respectively. In terms of treatment protocols, 1 patient (1.9%) received pure radiation therapy, 8 patients (15.38%) received pure chemotherapy, 40 patients (76.92%) received surgery + chemotherapy and 3 patients (5.7%) received chemotherapy + surgery + radiation (Figure 4). Patients who survived more than 5 years were all treated with surgery oriented comprehensive therapies: surgery + chemotherapy or chemotherapy + surgery + radiation. Of the chemotherapy regimens, 62.75% were based on bleomycin. On the choice of chemotherapy regimen, BEP regimen accounts for 54.90%, and VIP regimen 24.75%. The detailed clinical information is shown in Table 1.

**TABLE 1:** Summary of the clinical characteristics, treatments, and outcomes of the 52 patients with primary mediastinal yolk sac tumor.

Reference	Sex/age (years)	Histological type	Tumor size (cm)	Treatment protocols	Chemotherapy regimes	Status/follow‐up (months)
Akasbi^5^	M/26	YST	10 × 12	CS	BEP	Alive, 60
Al Ghamdi^6^	M/16	YST	NA	CS	PEB	Alive, 144+
Aroshidze^7^	M/20	YST	8.4 × 8.1 × 5.1	C	BEP, VIP	NA
Chaudhry^8^	M/18	YST	NA	CS	PEB	Alive, 60+
Darif^9^	M/19	MGCT	17	CS	VIP	Alive, 42+
Gkampeta^10^	M/3	YST	6.5 × 5.5	C	BEP	Alive, 48+
Hu X^11^	M/12	MGCT	NA	C	BEP, EP	Alive, 6+
Imaniar^12^	M/15	YST	15.2 × 15 × 12	R	‐	Died
Liu B^13^	M/16	YST	NA	CS	BEP	Alive, 38+
	M/17	YST	NA	CS	BEP, EP	Alive, 29+
	M/32	YST	NA	CS	BEP	Died, 11
	M/35	YST	NA	CS	BEP	Died, 5
	M/14	MGCT	NA	CS	BEP	Alive, 13+
	M/22	YST	NA	CS	EP	Alive, 5+
	M/22	YST	NA	CS	BEP	Alive, 3+
Mishra^14^	M/18	YST	NA	CS	VIP	Alive, 5+
	M/22	MGCT	NA	CS	VIP, TIP	Died, 18
Nakhla^15^	M/73	YST	14.5 × 7 × 6.6	C	VIP	Died
Noor^16^	M/22	YST	12	C	BIP	NA
Qin L^17^	M/30	YST	3.6 × 2.2 × 1.9	CS	EP	Alive, 57
	M/33	YST	10 × 7.5 × 6.7	CS	BEP	Died, 8
	M/29	YST	8.5 × 7.6 × 7.6	CS	EP	Alive, 31
	M/24	YST	5.6 × 6.3 × 6.5	CS	BEP	Alive, 25
	M/21	YST	2.6 × 3.8 × 1.2	CS	BEP	Alive, 46
	M/19	YST	6.7 × 8.2 × 7.5	CS	BEP	Alive, 61
	M28	YST	4.7 × 6.8 × 7.1	CS	BEP	Died, 12
	M/22	YST	6.5 × 6.3 × 7.5	CS	BEP	Died, 7
	M/29	YST	7.5 × 6.3 × 5.5	CS	EP	Died, 15
	M/31	YST	11 × 4.7 × 7.5	CS	BEP	Alive, 36
	M/32	YST	5.8 × 5.6 × 8.0	CS	BEP	Alive, 22
	F/30	YST	6.8 × 6.5 × 7.2	CS	BEP	Died, 11
	M/34	YST	5.6 × 6.1 × 7.2	CS	EP	Alive, 28
	M/25	YST	7.6 × 5.6 × 6.2	CS	BEP	Died, 6
	M/22	YST	10.8 × 7.6 × 6.4	CS	BEP	Alive, 44
Sakaguchi^18^	F/60	YST	4.5 × 3	CSR	Bleomycin	Alive, 72+
Sakakura^19^	M/21	MGCT	14 × 10	CS	BEP	Alive, 12+
Silva^20^	M/38	YST	14.6 × 11.6 × 9.1	CS	BEP, TIP	NA
Soriano^21^	M/24	YST	NA	C	VIP, EP	NA
Sudour^22^	F/1	YST	NA	CS	VIP	Alive, 156
	F/1	YST	NA	CS	VIP	Alive, 48
	F/3	YST	NA	CSC	VIP	Alive, 108
	M/14	MGCT	NA	CSC	VIP, PV	Alive, 144
	M/14	YST	NA	CSC	VIP	Alive, 96
	M/17	MGCT	NA	CS	VIP	Alive, 12
Tanaka^23^	M/25	YST	7.6 × 6.5 × 3.5	CS	BEP, EP	Alive, 36+
	M/22	YST	14.4 × 9.8 × 10	CS	BEP, EP	Alive, 16+
Yalçın^24^	M/2.5	YST	NA	CS	VACA	Died, 4
	M/16	YST	NA	CSR	BEP	Died, 19
	F/2	YST	NA	CSR	VAC	Alive, 284+
	M/11	YST	NA	C	BEP	Died, 3
Yang Q^25^	M/60	YST	NA	C	VIP	Alive, 6+
Zhu F^26^	M/42	YST	10 × 8	CS	BEP, VIP	NA

Abbreviations: BEP, bleomycin, etoposide, and cisplatin; BIP, bleomycin, ifosfamide, cisplatin; C, chemotherapy; CS, chemotherapy + surgery; CSR, chemotherapy + surgery + radiation; EP, etoposide, cisplatin; F, female; M, male; MGCT, mixed germ cell tumor; NA, not available; PEB, paclitaxel, etoposide, bleomycin; PV, cisplatin and etoposide; R, Radiation; TIP, paclitaxel, ifosfamide, cisplatin; VIP, etoposide, ifosfamide, and cisplatin; YST, yolk sac tumor; “+” means longer than record time.

## 4 DISCUSSION

Because of the low incidence of PMYST, most of the literature were merely limited in sporadic case reports. No accepted staging criteria and treatment guidelines could be available by far. The adopted staging method in clinical practice usually refers to that of thymoma,^27^ and the conventional treatment methods for PMYST is surgical resection and/or chemotherapy. According to our literature review, 76.79% of PMYST patients received surgery + chemotherapy.

Undoubtedly, surgical therapy plays an important role in the treatment of PMYST. Both single‐center and multi‐center reports have indicated that tumor resection may provide long‐term survival and cure opportunities for patients.^2^, ^17^, ^28^ The surgical approach included median sternotomy, lateral thoracotomy and hemi‐mussel incision, which was usually according to the location, size and extent involved by the tumor, as well as the basic condition of the patient. Extended excision may benefit more for patients in whom the tumors have invaded surrounding tissues and/or organs, such as lung, pericardium, diaphragm, and so on. Yang et al. also reported that when compared surgery with chemotherapy and radiotherapy, the long‐term survival rate was significantly improved in patients who underwent surgery, especially in those of radical resection.^2^ For patients with large vessels involved, we believe that resection and reconstruction of the vessels is also advisable. But the decision of using CPB requires careful trade‐offs because of the potential risk of tumor metastasis carried by CPB itself. Sakakura et al. shared a case that the primary mediastinal nonseminomatous germ cell tumor, damaging to the pulmonary trunk adjacent to the right ventricular outflow tract in a 21‐year‐old male, resulted in life‐threatening massive bleeding for the absence of cardiovascular surgeons during operation. Therefore, they presented some constructive consensus including cardiovascular surgeons should be consulted in advance when handling the area around great vessels and the use of an assisted circulation can be considered for safety reasons.^19^ As far as our case is concerned, although the CPB was not used, the surgical plan considering all the possibilities which may be encountered in operation was predesigned, including the use of CPB, and prosthetic vessel replacement. As far as current studies are concerned, there is no standardized surgical protocol for PMYST. In our literature review, no significant difference about surgical scope and incision margin in the surgical rate was found. It is possibly related to the sample size and postoperative adjuvant therapy. However, for malignant tumors, the radical excision with no tumor invasion at resection margin should be guaranteed as far as possible. Necchi et al. reported that the negative margin of radical surgery is a protective factor for the prognosis of patient with primary mediastinal nonseminomatous germ cell tumors.^29^

Non‐surgical methods, including chemotherapy and radiotherapy, are advisable for the adjuvant treatment of surgery and unresectable tumors. At present, the chemotherapy regimens of YST involved BEP, VIP (etoposide, ifosfamide, and cisplatin), EP (etoposide and cisplatin), which are used alone or in combination. On the choice of chemotherapy regimen, 54.90% of PMYST patients received BEP regimen and 24.75% received VIP regimen. BEP regimen was once the preferred choice for primary mediastinal nonseminomatous germ cell tumors (PMNGCTs). Mikhail et al. reported that BEP regimen or BEP‐based improved chemotherapy regimen was significantly associated with better outcome for mediastinal nonseminomatous germ cell tumors.^30^ Our literature review also showed that 62.75% chemotherapy regimens for PMYST were based on bleomycin. But, in recent years, many studies had pointed out that bleomycin has pulmonary toxicity contributed to pulmonary complications after surgery.^28^, ^30^ Meanwhile, the VIP regimen, as another alternative of chemotherapy, was reported to have the similar efficacy to BEP, and it is more suitable for patients who are awaiting surgery because of the lower pulmonary toxicity. However, similar to the case in our report, some studies have showed that BEP and VIP have no significant effect on tumor size.^6^, ^15^, ^19^, ^20^ To date, opinions on the necessity of chemotherapy and the selection of chemotherapy scheme, still remains controversial.

In addition, studies about radiotherapy of PMYST were also reported in limited articles.^12^, ^18^, ^24^ In this literature review, only one patient (1.9%) received radiation therapy alone.^12^ It is difficult to assess the efficacy of radiotherapy. But the prognosis of this patients was discouraging. He is 15‐year‐old male patient of PMYST with superior vena cava syndrome. No evidence of tumor atrophy was found, and the patient died soon after radiotherapy, lost the chance for further chemotherapy or surgery.^12^ Bilgehan et al. reported that combined treatment of radiotherapy together with surgery and chemotherapy provided more than 284 months survival for a 2‐year‐old female patient.^24^ Nevertheless, Yang et al. reported that the efficacy of chemotherapy + surgery is equivalent to that of surgery alone. Radiotherapy or chemotherapy showed no survival benefit.^2^ Radiotherapy and chemotherapy may be an important adjunctive therapy for PMYST, but its efficacy remains to be further verified.

Our literature review indicated that surgery oriented comprehensive therapies are the main treatment methods of PMYST. Surgery + chemotherapy is the most common selection. Although most surgeons strive for complete tumor removal, there is no evidence of a relationship between surgical R0 resection and survival benefit. Interestingly, three patients who received chemotherapy + surgery + radiotherapy appeared to have better clinical outcomes, although the tumor relapsed soon after surgery.^18^, ^24^ One of them survived for 19 months, and the other two lived for more than 5 years.

According to this literature reviewed and the treatment experiences of our case, surgery combined with chemotherapy is an important treatment modality for PMYST, although there is no unified and standardized treatment regimen. For the treatment strategies, the pathological components, the size, location, and degree of invasion of the tumor should be fully considered, thus the individualized treatment regimen could be made. Especially for patients of incomplete tumor resection or postoperative recurrence, a combination therapy of chemotherapy, surgery, and radiation may help patients achieve longer survival.

Apart from the treatment regime, other factors affecting prognosis should be considered. A study reported that age ≥24 years and size of the tumor ≥19 cm were found as independent negative prognostic factors in patients with mediastinal nonseminomatous germ cell tumors.^31^ Although it has not been confirmed, gender may be another important factor. We found that the prevalence of female MGCT is lower and female patients tends to have better prognoses. The research is limited due to the small sample size and heterogeneity of various studies. The prognostic factors of PMYST still need to be clarified by large‐scale multicenter prospective studies.

## 5 CONCLUSION

In summary, at present, due to the rarity of PMYST, no accepted staging criteria, and treatment guidelines could be available. The surgery oriented comprehensive therapies are still the main treatment methods. The surgical strategy should aim at radical resection and be designed individuality, considering all the possibilities during operation, including the use of cardiopulmonary bypass, and prosthetic materials.

## AUTHOR CONTRIBUTIONS

**Diqing Wu:** Data curation; writing – original draft. **Kun Zhang:** Resources; writing – original draft. **Xueqin Zhang:** Conceptualization; formal analysis; methodology; project administration; resources; supervision; writing – review and editing.

## FUNDING INFORMATION

The authors received no financial support for the research.

## CONFLICT OF INTEREST STATEMENT

Diqing Wu and Kun Zhang have equally contributed to this work. All authors have no conflict of interest or financial ties to disclose.

## ETHICS STATEMENT

The publication of this paper and any accompanying pictures has obtained the written informed consent of the patient's guardian. Ethical approval all procedures performed in studies involving human participants were in accordance with the ethical standards of the institutional and/or national research committee and with the 1964 Helsinki Declaration and its later amendments or comparable ethical standards.

## CONSENT

Written informed consent has been obtained from the patient's parents to publish this report in accordance with the journal's patient consent policy.

## Data Availability

All data generated or analyzed in this study are included in this article.
